# Supplementary material for: Random bit generation based on a self-chaotic microlaser with enhanced chaotic bandwidth
Source: Nanophotonics. 2023 Oct 13;12(21):4109–16. doi: 10.1515/nanoph-2023-0549 (PMC11502037; doi:10.1515/nanoph-2023-0549)
Supplement: Supplementary file 1 — Supplementary Material Details [file j_nanoph-2023-0549_suppl_001.pdf]

Supplementary Materials

Random bit generation based on a self-chaotic microlaser with enhanced chaotic bandwidth

Jian-Cheng Li[1,2], Jin-Long Xiao[1,2], Yue-De Yang[1,2], You-Ling Chen[1,2], and Yong-Zhen Huang[1,2*]

1. State Key Laboratory of Integrated Optoelectronics, Institute of Semiconductors, Chinese Academy of Sciences, Beijing 100083, China
2. Center of Material Science and Optoelectronic Technology, University of Chinese Academy of Sciences, Beijing 100049, China
* Correspondence author: yzhuang@semi.ac.cn.

Section 1: Optimization for the deformed circular-sided square microcavity

Mode characteristics of the circular-side square microcavity with a center hole are numerically investigated by a two-dimensional (2D) finite element method (FEM, via software COMSOL Multiphysics 5.0). Figure S1 shows the simulation schematic diagram, where *a*, *r*, *d*, and *h* are the flat-side length of the square, the radius of the circular arc, the width of output waveguide and the shift distance of the output waveguide, respectively. The circular-side deformation parameter $\delta = r - \sqrt{r^2 - a^2/4}$, and the radius of the circle hole R_{in} are specially optimized to control mode intervals and mode numbers. In the numerical simulation, the refractive indices of cavity and surrounding bisbenzocyclobutene (BCB) material are set to 3.2 and 1.54, respectively. The perfectly matched layer (PML) with a width of 1 μm is used to terminate the simulation window with a distance to the microcavity larger than 7 μm.

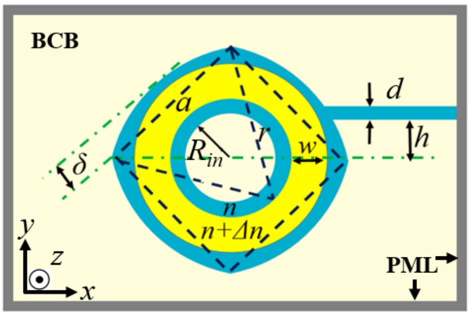

Fig. S1. Schematic diagram of the deformed square microcavity with a center hole for 2D FEM simulation.

For the circular-sided square cavity with a = 20 μm, d = 1.5 μm, and $h = R_{in} = 0$, the calculated mode Q-factors and mode frequency interval Δf_{01} between the fundamental transverse mode and the first-order transverse mode versus the deformation δ are plotted in Fig. S2(a). The mode Q-factors for the fundamental, first-order mode and second-order transverse modes and the mode interval Δf_{01} decrease firstly and increase again, as δ increases from 2.10 to 2.23 μm. The value of Δf_{01} is below 10 GHz as δ ranged from 2.17 to 2.21 μm. We selected δ = 2.17 μm for further optimization. The mode Q-factors of the 0th, 1st, and 2nd transverse modes under the selected deformation parameter are 1.7×10^7, 2.4×10^6 and 6×10^5, which are all larger than 10^4 and indicate the possible lasing of more transverse modes. Furthermore, a central circular hole is introduced to control the number of transverse modes [1]. As shown in Fig. S2(b), the Q-factors for three low-order modes remain almost unchanged as $R_{in} < 3$ μm because of their nearly zero field distributions in the center of the cavities, and then decrease rapidly as $R_{in} > 3$ μm due to the increasing overlap between the hole and mode fields. The more overlap, the stronger destruction of the mode field distributions and the fewer transverse modes exist. When R_{in} reaches 7 μm, the Q-factor for 2nd transverse mode is about 1.4×10^3. The mode interval Δf_{01} remains 7 GHz as R_{in} is below 6.5 μm, and then increases rapidly when R_{in} is larger than 6.5 μm. Accounting the tradeoff of mode interval and mode numbers, we choose R_{in} = 5.5 μm for three transverse modes lasing. By shifting the output waveguide, we can attain higher output coupling efficiency. Figure S3 shows the influence of the output waveguide position on the mode Q-factors and output coupling efficiencies. When $h = 4\sqrt{2}$ μm, the maximum coupling efficiencies for 0th, 1st, and 2nd modes can be obtained simultaneously, and the mode Q-factors and mode interval are little affected by the output waveguide yet. Finally, accounting the magnetic field ($|H_z|$) distributions for 0th, 1st, and 2nd modes in Fig. 1(c), we design a ring p-electrode with a width of 4 μm and an inner radius of 6.5 μm for precisely controlling the transverse mode interval and lasing mode numbers.

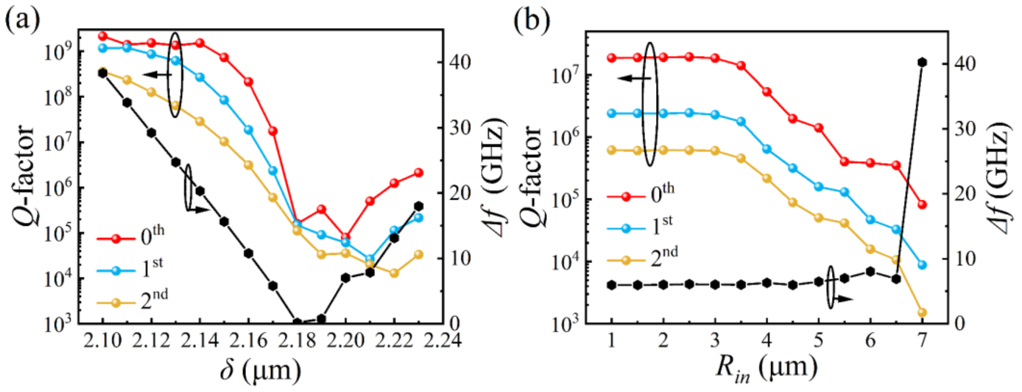

Fig. S2. Mode Q-factor and mode interval Δf_{01} versus (a) the circular-side deformation δ, and (b) the radius of central hole R_{in}.

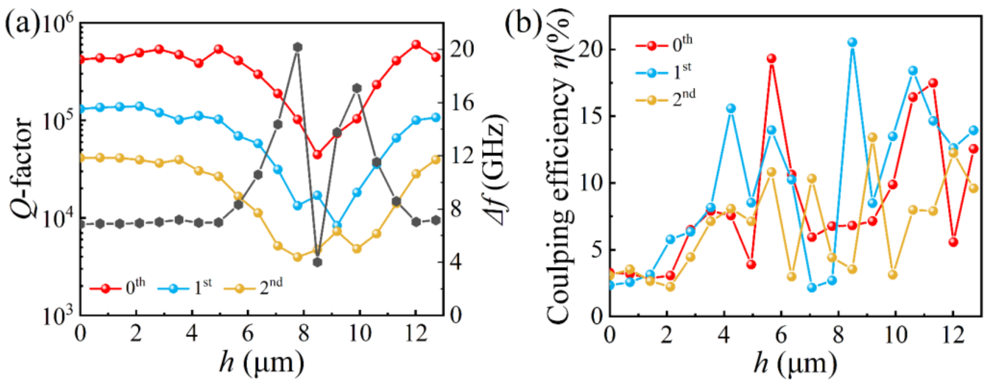

Fig. S3. (a) Mode Q-factor, mode interval Δf_{01}, and (b) coupling efficiency versus output waveguide shift distance h.

Section 2: Correlation dimension estimation

Chaos attractor can be characterized by fractal dimension (nearly equal to correlation dimension). To reduce the influence of noise and improve the calculation efficiency of correlation dimension, a modified Grassberger-Procaccia (GP) algorithm is adopted, using re-embedding procedure based on singular-value decomposition technique [2]. In the d-dimensional re-embedding space, correlation integral C_d (r) denotes the average number of points inside a ball of radius r centered at one point. The correlation dimension D is estimated from the correlation integral curve by

$$D = \lim_{\substack{d \to \infty \\ r \to 0}} \frac{d \ln(C_d(r))}{d \ln(r)}$$

Here, the experimentally temporal data of 4000 points are utilized to estimate D, with window size of 20 and five principal components in procedure. The logarithmic correlation integral C_d (r) versus logarithmic r and corresponding slope of $\ln(C_d$ $(r))$

are presented in Figs. S4(a) and S4(b), respectively, for d = 15, 16, 17 and 18. A correlation dimension of 11.6 is estimated from flat plateau scaling region in Fig. S4(b).

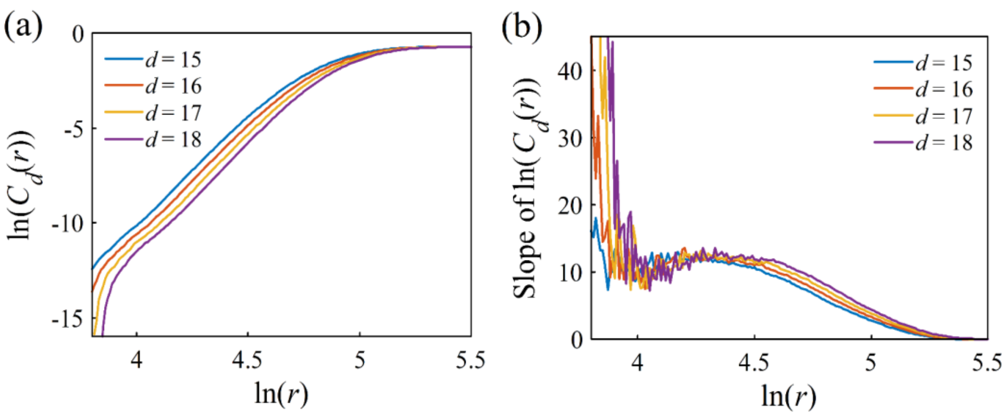

Fig. S4. The correlation integral C_d (r) versus the radius of the ball in logarithmic scale, and (b) corresponding slope of ln(C_d (r)) curve.

References

[1] Liu X. W. Ma, X. M. Lv, Y. Z. Huang, Y. D. Yang, J. L. Xiao, and Y. Du," Mode characteristics for unidirectional-emission microring resonator lasers," J. Opt. Soc. Am. B, vol. **31**, pp. 2773-2778, 2014.

[2] K. Fraedrich and R. H. Wang, "Estimating the correlation dimension of an attractor from noisy and small datasets based on re-embedding," Phys. D, vol. **65**, pp. 373-398, 1993.
